# Endoscopic Transnasal and Transclival Resection of Cholesteatomas in the Clivus and Temporal Bone Pyramid: A Case Series of Eight Patients

**DOI:** 10.7759/cureus.77330

**Published:** 2025-01-12

**Authors:** Alexey N Shkarubo, Ilya V Chernov, Vladislav V Ivanov, Diana S Adueva, Elen L Pogosyan

**Affiliations:** 1 Neurooncology, NN Burdenko National Medical Research Center of Neurosurgery, Moscow, RUS; 2 Center for Health Information Technology (IT) and Social Medicine, Sechenov University, Moscow, RUS

**Keywords:** cholesteatoma, clivus, endoscopic transnasal neurosurgery, skull base, temporal bone pyramid

## Abstract

Introduction

Cholesteatomas of the clivus and pyramids of the temporal bone can significantly reduce the quality of life of patients due to damage to the cranial nerves. The only effective method for their treatment is surgery.

Materials and methods

The study included a case series of eight patients: five females and three males. In all cases, the lesions were located within the clivus and pyramid of the temporal bone. All operations were performed using an endoscopic transnasal approach.

Results

In all cases, the patients underwent successful surgical treatment. Near-total removal (95%-99% of the neoplasm) was accomplished in all eight cases. Among complications, only postoperative cerebrospinal fluid (CSF) leakage was noted, which occurred in one (12.5%) patient.

Conclusions

Thanks to the development of endoscopic technologies, it has become possible to safely remove cholesteatomas of the clivus and pyramid without extensive trepanation and traumatic translabyrinthine approaches.

## Introduction

Cholesteatomas are tumor-like masses composed of desquamated epithelium, originating from the ectodermal germ layer. They can be classified into congenital and acquired forms, the latter arising as a result of chronic infection or surgical interventions [1,2]. Generally, cholesteatomas arise in the middle ear behind an intact tympanic membrane [3]. Less frequently, cholesteatomas are found within the pyramid of the temporal bone, accounting for 4% to 9% of all pyramidal lesions. Even more rarely, cholesteatomas can extend from the pyramid to the clivus [4]. In such cases, cholesteatomas begin to compress the adjacent cranial nerves, potentially leading to complete nerve dysfunction. This results in a progressive loss of hearing, facial nerve paralysis, and oculomotor disturbances [5]. Computed tomography imaging of cholesteatomas typically reveals a hypodense signal along with destruction of the bony structures of the skull base. Magnetic resonance imaging findings display a hyperintense signal in T2-weighted images, as well as a signal similar to that of cerebrospinal fluid in T1-weighted sequences [6-8]. The only treatment option for cholesteatomas of the pyramid and clivus is surgical intervention. Depending on the neoplasm's location, either a trans-pyramidal or an endoscopic transnasal approach may be warranted. The choice of surgical approach can be guided by the classification of cholesteatomas proposed by Sanna et al. [9], which categorizes them into five types: supralabyrinthine, infralabyrinthine, infralabyrinthine-apical, massive, and apical. For cholesteatomas located in the medial sections of the temporal bone pyramid, the use of an endoscopic transnasal approach is anatomically justified, although there is limited literature describing this technique [3,10-13]. In this report, we share our experience with the endoscopic transnasal removal of cholesteatomas localized to the temporal bone pyramid and the clivus.

## Materials and methods

The study included eight patients; five female and three male patients.

Inclusion criteria

The study included patients with cholesteatomas of the clivus and petrous bone verified by preoperative CT and MRI of the brain according to the classification of Sanna et al. [9] who underwent endoscopic transnasal transclival surgery at the Burdenko National Medical Research Center for Neurosurgery, Moscow, Russia.

Sanna's classification of petrous bone cholesteatomas includes five classes of these lesions: supralabyrinthine, infralabyrinthine, infralabyrinthine-apical, massive (lesion spreads to the entire pyramid and clivus), and apical cholesteatoma [9].

Exclusion criteria

Patients with cholesteatomas of the clivus and petrous bone who underwent transcranial surgical interventions at the same institution were excluded.

Study process

All patients were diagnosed with clivus and temporal bone pyramid cholesteatomas and underwent surgical treatment at the Burdenko National Medical Research Center for Neurosurgery from 2015 to 2024. Prior to the surgery, all patients underwent an MRI of the brain with contrast enhancement, which allowed them to have a suspicion of cholesteatoma. The clinical manifestations included visual disturbances, oculomotor dysfunction, facial sensory deficits, facial asymmetry due to facial nerve paresis, and hearing loss.

Surgical procedure

Given the tumors’ localization predominantly in the medial sections of the skull base (clivus, medial, and middle sections of the temporal bone pyramid), we propose that the endoscopic transnasal transsphenoidal approach is the optimal surgical technique. All interventions were conducted using the Karl Storz surgical endoscopic system (Karl Storz SE & Co. KG, Tuttlingen, Germany). During tumor excision, a variety of endoscopes (0°, 30°, 45°, and 70°), angled curettes, forceps, and suction devices were utilized. We have progressively reduced our reliance on external lumbar drainage, with the last five patients in the series undergoing the procedure without its use.

The surgical steps are as follows: 1) access the sphenoid sinus; 2) clivus trepanation if it is not affected by the lesion; 3) opening of the lesion's capsule; 4) removal of the lesion (the difficulty of this step lies in working directly next to the internal carotid artery); 5) hemostasis; 6) closure of the skull base defect using artificial or auto materials. In one case of intraoperative cerebrospinal fluid (CSF) leakage, closure of the defect was accomplished using autologous tissues and fibrin glue.

## Results

The median age of patients was 54 years (range: 32-64 years). In all cases, the tumors originated within the clivus and pyramid of the temporal bone; in six cases, the cholesteatomas extended into the clivus, while in two cases, they invaded the sphenoid sinus and posterior sections of the ethmoid bone. Notably, in all cases, lesions were situated extradurally.

Visual disturbances were found in two patients (25%), and oculomotor dysfunction in four patients (50%, including involvement of the cranial nerve III in one patient and cranial nerve VI in three patients). Sensory deficits were observed in six patients (75%), while facial asymmetry due to facial nerve paresis was observed in four patients (50%), with a degree of impairment of at least four points according to the House-Brackmann scale. Additionally, hearing loss was observed in two patients (25%), attributed to the involvement of the auditory nerve within the pyramid of the temporal bone. 

In our opinion, achieving complete removal of a cholesteatoma is impossible. Therefore, we consider a visually complete resection, especially when small fragments only remain near the carotid artery or temporal bone, to be near-total removal (95%-99% of the lesion) [14]. This was accomplished in all eight cases.

Clinical case number 1

**Figure 1:**
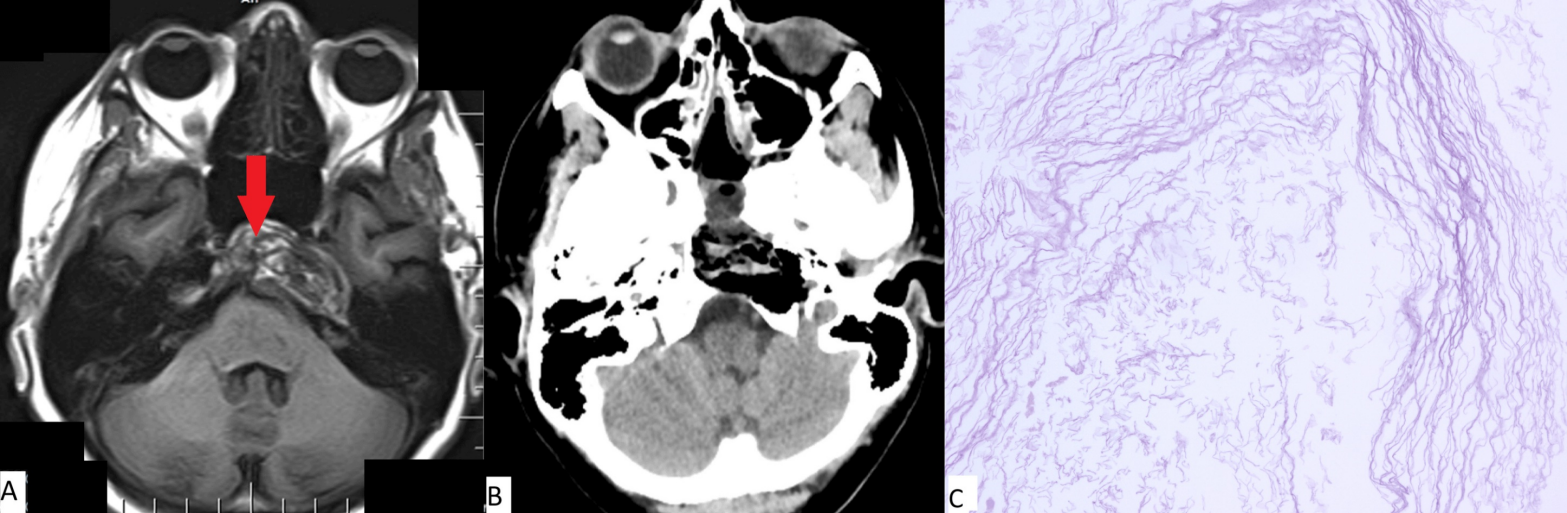
(A) MRI prior to surgery; (B) CT obtained immediately after surgery for patient 1; (C) Histopathologic image (H&E, x100) Complete removal of the cholesteatoma (red arrow in pane A) of the left temporal bone pyramid and clivus is observed. Typical cholesteatoma is noted in pane C.

The patient was a 58-year-old who was admitted to our institution with complaints of diplopia. In January 2012, the patient underwent surgical removal of an epidermoid cyst at the apex of the pyramid of the temporal bone on the left. In October 2018, she complained of signs of diplopia. An MRI study showed a relapse of the epidermoid cyst (Figure 1). In February 2019, endoscopic transnasal removal of a large cholesteatoma of the clivus and pyramid of the left temporal bone was performed. The postoperative period was complicated by a CSF leak, leading to a revision surgery. The patient was discharged in a satisfactory condition on the 20^th^ day after surgery.

Clinical case number 2

**Figure 2:**
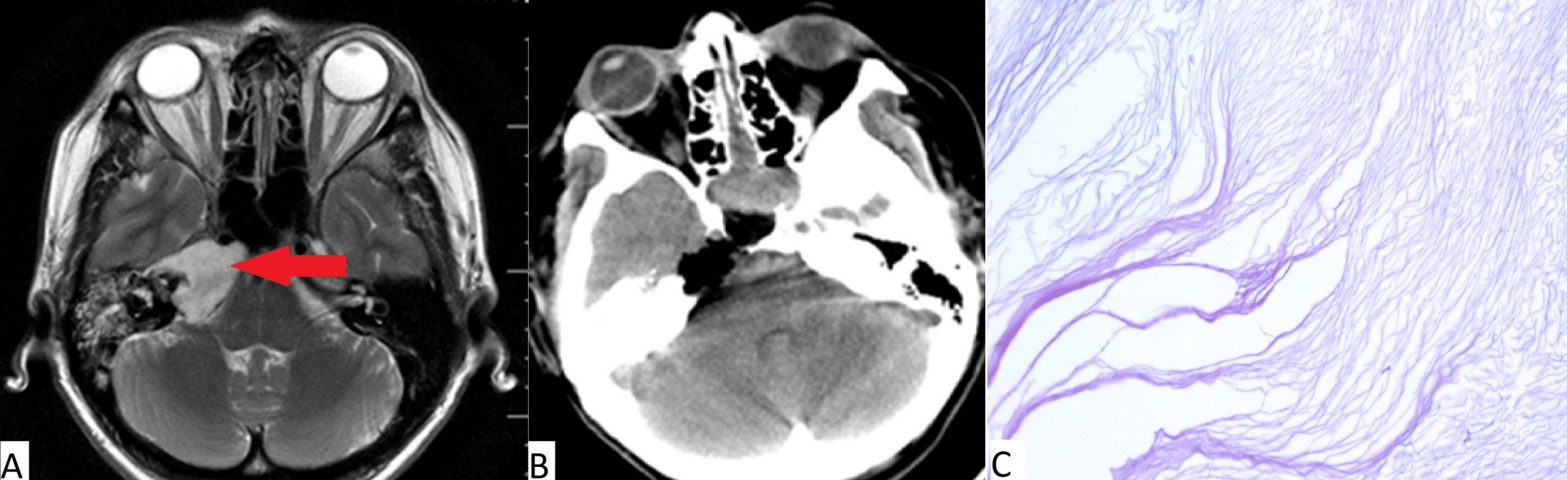
(A) MRI prior to surgery; (B) CT obtained immediately after surgery for patient 2; (C) Histopathologic image (H&E, x100) Complete removal of the cholesteatoma (red arrow in pane A) of the left temporal bone pyramid is observed. Typical cholesteatoma is noted in pane C.

The patient was a 59-year-old who was admitted to our institution with complaints of facial asymmetry and diplopia. According to the patient, facial asymmetry was first noticed 30 years prior. An MRI of the brain revealed a lesion in the area of the pyramid of the right temporal bone (Figure 2). Neurological status was as follows: decreased facial sensitivity on the right along the three branches of cranial nerve V, paresis of the facial nerve on the right, and lagophthalmos. Visual acuity (with correction) was as follows: right eye (OD) = 0.7, left eye (OS) = 0.8. Severe paresis of cranial nerve VII, paresis of cranial nerve VI, insufficiency of cranial nerve V, and trophic keratopathy were noted. In July of 2024, an endoscopic transnasal removal of a lesion of the pyramid of the right temporal bone was performed. The postoperative period was uneventful. No deterioration of the neurological status after the surgery was observed. The patient was discharged on the sixth day after surgery.

Clinical case number 3

**Figure 3:**
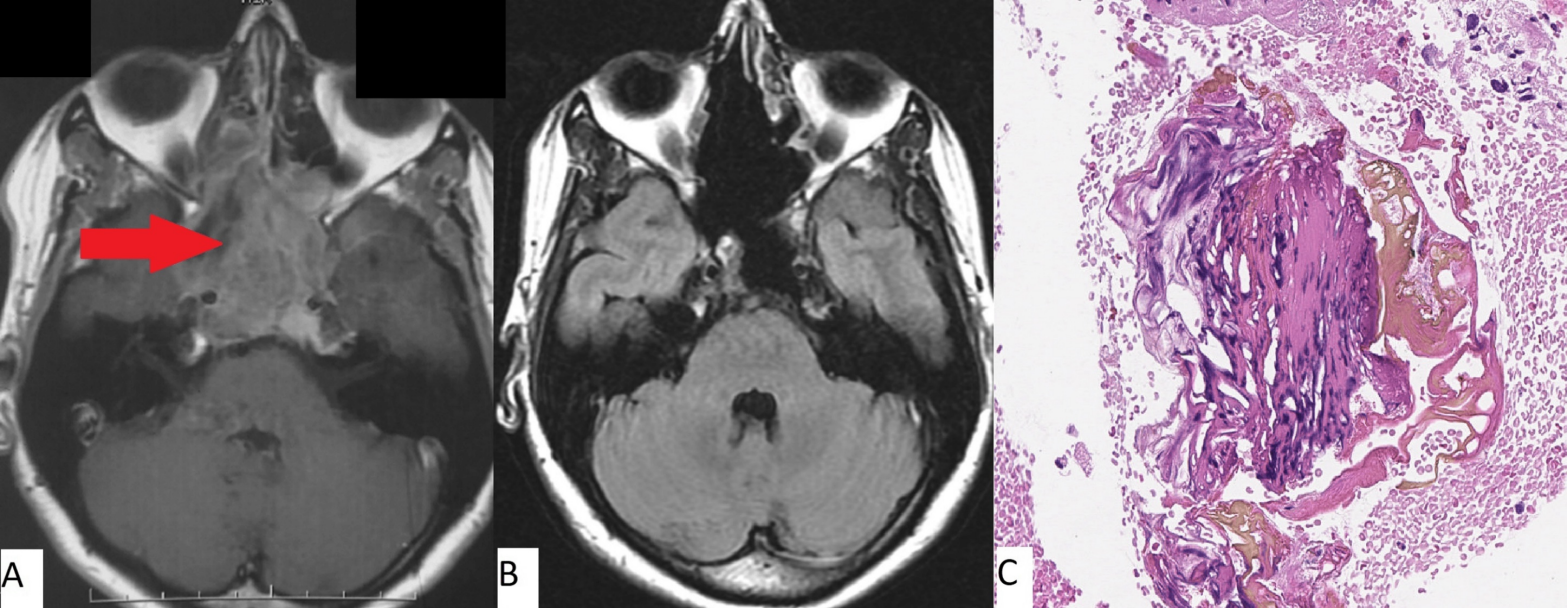
MRI before surgery (A) and 3.5 years postoperatively (B) for patient 3 ; C: Histopathologic image (H&E, x100) Complete removal of the cholesteatoma of the sphenoid sinus, clivus, and the apices of both temporal bone pyramids (red arrow in pane A) is observed. Typical cholesteatoma is noted in pane C.

A 35-year-old was admitted to our institution with complaints of impaired sensitivity in the right half of the face. An MRI of the brain revealed a large lesion of the skull base (Figure 3). In July 2015, an endoscopic transnasal removal of a large lesion of the skull base was performed. In the postoperative period, neurological improvement was noted in the form of regression of hypoesthesia of the first and second branches of cranial nerve V on the right. The patient was discharged in satisfactory condition on the eighth day after surgery.

Clinical case number 4

**Figure 4:**
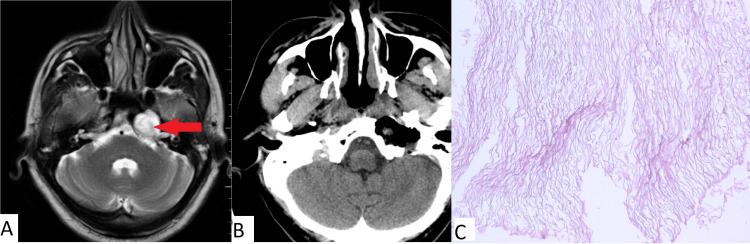
(A) MRI prior to surgery; (B) CT obtained immediately after surgery for patient 4; (C) Histopathologic image (H&E, x100) Complete removal of the cholesteatoma (red arrow in pane A) of the left temporal bone pyramid is observed. Typical cholesteatoma is noted in pane C.

A 32-year-old patient was admitted to our institution with complaints of diplopia with an onset in September 2021. An MRI of the brain revealed a large lesion in the region of the clivus and the pyramid of the left temporal bone (Figure 4). During an examination by an ophthalmologist, dysfunction of cranial nerve VI on the left was reported. In December 2021, an endoscopic transnasal removal of an extensive neoplasm of the pyramid of the left temporal bone was performed. The postoperative period was uneventful. The postoperative neurological status was without deterioration. During an examination by an ophthalmologist, the degree of visual impairment was unchanged. Partial regression of the insufficiency of the left abducens nerve was reported. The patient was discharged on the seventh day after surgery in satisfactory condition.

Clinical case number 5

**Figure 5:**
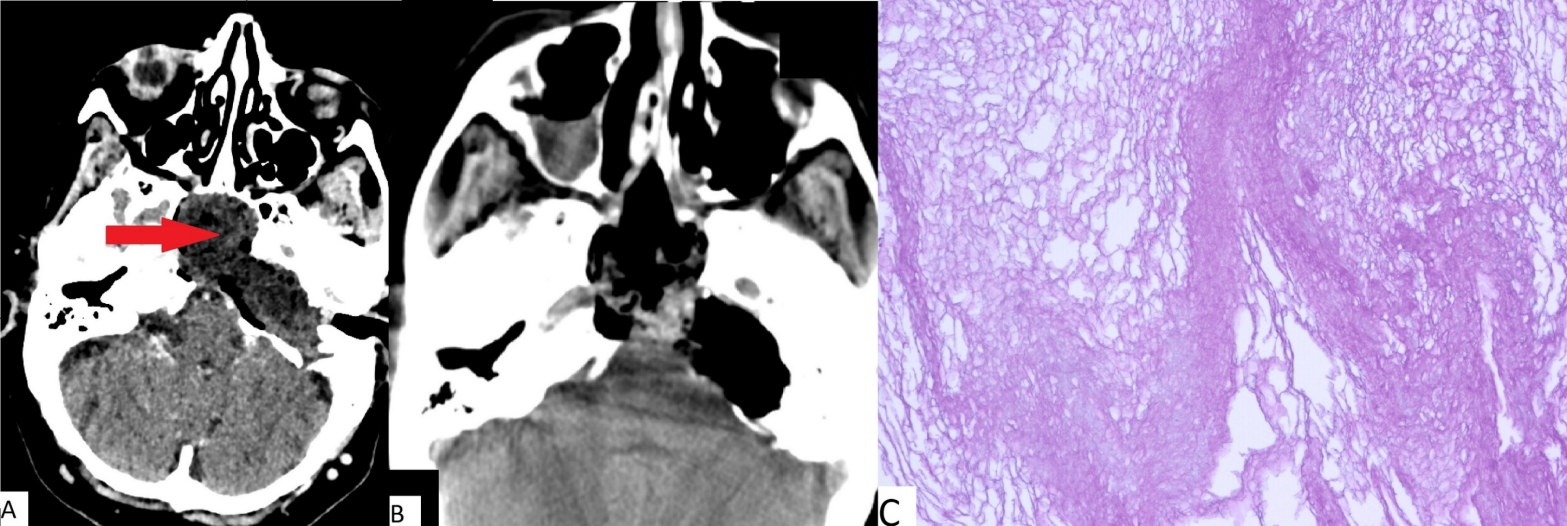
(A) MRI prior to surgery; (B) CT obtained immediately after surgery for patient 5; (C) Histopathologic image (H&E, x100) Complete removal of the cholesteatoma of the sphenoid sinus, clivus, and the left temporal bone pyramid (red arrow in pane A) is observed. Typical cholesteatoma is noted in pane C.

A 64-year-old was admitted to our institution with complaints of headache, periodic CSF leaks, and blindness in the left eye. In 1989, the disease manifested in the form of peripheral paresis of the left facial nerve. The patient noted periodic CSF leakage (nasal, auricular). At examination, a large cholesteatoma of the clivus and pyramid of the left temporal bone was detected (Figure 5). In February 2024, endoscopic transnasal removal of a large cholesteatoma of the clivus and pyramid of the left temporal bone was performed. The postoperative period was uneventful. No postoperative deterioration in the neurological status was observed. The patient was discharged on the seventh day after surgery in satisfactory condition.

Clinical case number 6

**Figure 6:**
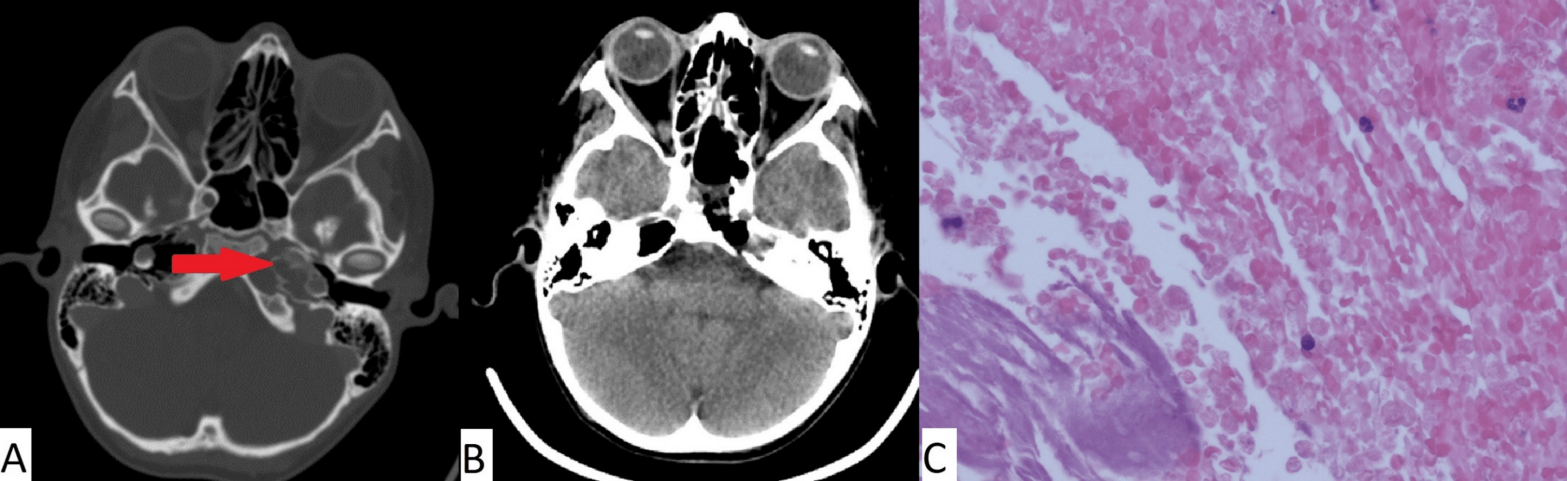
CT obtained before surgery (A) and immediately after surgery (B) for patient 6; C: Histopathologic image (H&E, x400) Complete removal of the cholesteatoma of the sphenoid sinus, clivus, and the left temporal bone pyramid  (red arrow in pane A) is observed. Typical cholesteatoma is noted in pane C.

The patient was an 11-year-old who was admitted to our institution with complaints of diplopia, headaches, and ringing in the left ear. An MRI examination demonstrated a lesion destroying the apex of the pyramid of the temporal bone on the left with MR characteristics of a cholesteatoma (Figure 6). In July 2022, an endoscopic transnasal transclival removal of an epidermoid cyst of the pyramid of the left temporal bone was performed. The postoperative period was uneventful. The neurological status remained unchanged in relation to the preoperative state. The patient was discharged on the fifth day after surgery in satisfactory condition.

Clinical case number 6

**Figure 7:**
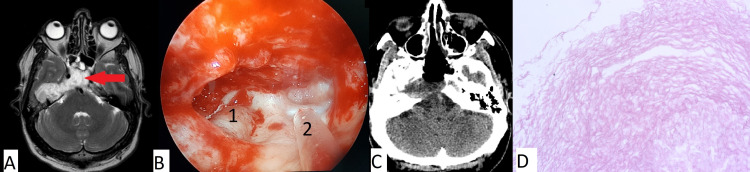
(A) MRI; (B) intraoperative photo which shows the pyramid of the temporal bone; (C) CT after surgery for patient 7; D: Histopathologic image (H&E, x100) Large cholesteatoma of the clivus and pyramid of the right temporal bone (red arrow in pane A) removed. In pane B, 1 indicates the internal carotid artery (petrosal segment), and 2 indicates the suction tube. Pane D indicates a typical cholesteatoma.

A 50-year-old was admitted to our institution with complaints of headaches, decreased hearing in the right ear, paresis of the right half of the face, and signs of meningitis. During the examination, an MRI of the brain revealed a large lesion of the clivus and right temporal bone (Figure 7). In March 2019, an endoscopic transnasal removal of a giant cholesteatoma of the clivus and right temporal bone was carried out. The postoperative period was uneventful. There were no negative changes in the neurological status. The patient was discharged on the fifth day after surgery in satisfactory condition.

Clinical case number 8

**Figure 8:**
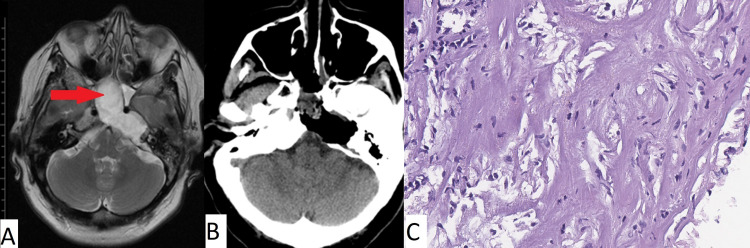
A) MRI prior to surgery; (B) CT obtained immediately after surgery for patient 8; (C) Histopathologic image (H&E, x100) Complete removal of the cholesteatoma of the sphenoid sinus, clivus, and the left temporal bone pyramid (red arrow in pane A) is observed. Typical cholesteatoma is noted in pane C.

The patient was a 51-year-old who was admitted to our institution with complaints of headache, discomfort in the left half of the face, severe hearing loss in the left ear, and inability to close the left eyelid. According to the patient’s history, these complaints have persisted for approximately 10 years. The MRI data revealed an apical cholesteatoma of the left temporal bone pyramid extending to the clivus (Figure 8). An endoscopic transnasal removal of the neoplasm was performed. A control CT scan after the surgery demonstrated complete removal of the epidermoid cyst and no air in the cranial cavity. The patient was discharged in a satisfactory condition seven days later.

The duration of the surgical interventions varied from 120 to 220 minutes, with a median of 160 minutes. Blood loss ranged from 50 to 100 ml. In all cases, patients were transferred to the postoperative recovery ward and later moved to the clinical ward. The patients’ return to activity was initiated on the first postoperative day. Most patients experienced a reduction in the severity of cranial nerve deficits, although complete recovery was not achieved in any of the cases by the time of discharge. Notably, there were no instances of neurological deterioration or new cranial nerve deficits during the postoperative period.

In terms of complications, only postoperative CSF leakage was observed in one (12.5%) patient (a small defect in the thinned underlying dura mater remained after lesion removal). The patient underwent a revision procedure for defect repair using autologous fat and fibrin glue.

In all cases, the diagnosis of cholesteatoma was histologically confirmed.

Patients stayed at our institution for a duration of five to 20 days, with a median length of stay of seven days. The longest stay was recorded for the patient who experienced postoperative CSF leakage. All patients were discharged in satisfactory condition.

Comprehensive follow-up data were available for all patients, with follow-up periods from one to four years. In all cases, no continued lesion growth was observed. The postoperative neurological status also remained stable without any changes.

The data for all cases are summarized in Table 1.

**Table 1:** Patient data for all cases

Patient details (age and sex)	Localization	Clinical manifestations	Intradural Growth	Radicality	Intraoperative complications	Postoperative CSF leakage	Catamnesis
58 years old, female	Pyramid of the left temporal bone	Left-sided paresis of cranial nerve VI	No	Near-total	Cerebrospinal fluid (CSF) leakage	Yes	1 year, no progression
59 years old, female	Pyramid of the right temporal bone	Severe paresis of cranial nerve VII, paresis of cranial nerve VI, insufficiency of cranial nerve V with trophic keratopathy development	No	Near-total	No	No	1 year, no progression
35 years old, female	Sphenoid sinus, clivus, pyramid of the temporal bone, and posterior parts of the ethmoid bone	Insufficiency of cranial nerve V	No	Near-total	No	No	2 years, no progression
32 years old, male	Clivus and left temporal bone pyramid area	Left-sided cranial nerve VI insufficiency	No	Near-total	No	No	1 year, no progression
64 years old, female	Sphenoid sinus, clivus, pyramid of the left temporal bone, and posterior parts of the ethmoid bone	Headache, recurrent CSF rhino- and otorrhea; functional loss of the left cranial nerve VIII, dysfunctions of cranial nerves V, VI, and VII. grade 3-4 right-sided auditory loss	No	Near-total	No	No	4 years, no progression
11 years old, male	Pyramid of the left temporal bone	Right-sided cranial nerve VI insufficiency	No	Near-total	No	No	2 years, no progression
50 years old, male	Clivus and the pyramid of the right temporal bone	Headaches, right-sided paresis of cranial nerve VII, insufficiency of cranial nerves V and VIII on the right side.	No	Near-total	No	No	3 years, no progression
51 years old, female	Clivus and the pyramid of the right temporal bone	Left-sided cranial nerve V insufficiency, left-sided facial nerve paresis, grade V (House- Brackman scale), and hearing loss on the left side	No	Near-total	No	No	4 years, no progression

## Discussion

The vast majority of patients diagnosed with cholesteatomas are treated by otolaryngologists. However, when the lesion extends intracranially or involves the structures of the skull base, including the clivus and the medial sections of the temporal bone pyramid, neurosurgical intervention becomes imperative. Advances in modern diagnostic techniques enable the accurate establishment of preoperative diagnosis and anatomical lesion characteristics, facilitating the planning of a tailored surgical approach in each specific case. Currently, various surgical strategies are employed for neoplasms localized in the middle cranial fossa, including traumatic transotic, transcochlear, and combined approaches to the infratemporal fossa [14]. Additionally, minimally invasive endoscopic transnasal techniques have gained traction, such as the standard transnasal transsphenoidal approach for cholesteatomas in the chiasmal and sellar region and the expanded posterior transclival approach for those located in the region of the clivus and temporal bone pyramid [15]. Cholesteatomas of the clivus and medial sections of the temporal bone pyramid are relatively rare [9, 16, 17]. The optimal surgical approach for their removal is the endoscopic transnasal transsphenoidal approach, despite the proximity of the internal carotid arteries within the surgical corridor. This approach allows for the least traumatic access to the clivus region and, consequently, the medial and middle sections of the temporal bone pyramid, without the need for visualization and traction of the facial and vestibulocochlear nerves, as required for the lateral approaches. Specialized micro-instruments facilitate manipulation within the area of the temporal bone pyramid extending up to its medial sections. Unfortunately, the most lateral sections of the pyramid remain largely inaccessible; thus, in such cases, a combined approach involving both the lateral and transnasal techniques may be warranted. Given the destructive nature of cholesteatoma expansion, the inner cortical plate of the affected bones is absent in many cases, leading to the exposure of the dura mater, which is closely adhered to the outer layers of the neoplasm. Consequently, achieving radical neoplasm resection is frequently associated with risks, including potential CSF leakage and damage to adjacent neurovascular structures (cranial nerves and vessels). Nonetheless, the layered and soft structure of the neoplasm permits its radical removal, which was successfully achieved in all presented cases. 

Unfortunately, the challenges of timely cholesteatoma diagnosis remain problematic, as patients frequently undergo MRI evaluation several years after the onset of clinical symptoms. Consequently, this delay often leads to gradual decompensation of cranial nerve functions, resulting in permanent function loss and a significant decline in the quality of life of the patients. Such irreversible neurological deterioration is illustrated by our series of patients, where even complete neoplasm resection did not lead to the restoration of critical neurological functions.

The limitations of this study are its single-center nature with limited sampling, which limits its demographic generalizability, as well as its descriptive design without comparison with a control group of patients operated on by other approaches.

## Conclusions

The views on surgical management of patients with cholesteatomas of the clivus and the temporal bone pyramid have reached a defined consensus. Clear indications for various approaches have been established based on the topographical and anatomical features of the tumor. The advancement and implementation of endoscopic transnasal surgical techniques have allowed for the most radical removal of these complex tumors with minimal complications, all while preserving the quality of life of the patients. It is advisable for such procedures to be performed in neurosurgical centers with extensive experience in microsurgical and endoscopic techniques targeting skull base structures. Nevertheless, the issue of timely diagnosis persists, primarily due to the late referrals of patients to specialized medical centers. 
